# Macrophages in Health and Non-Infectious Disease

**DOI:** 10.3390/biomedicines9050460

**Published:** 2021-04-23

**Authors:** Evgeny E. Bezsonov, Alexei Gratchev, Alexander N. Orekhov

**Affiliations:** 1 Laboratory of Angiopathology, Institute of General Pathology and Pathophysiology, 8 Baltiiskaya Street, 125315 Moscow, Russia; 2 Laboratory of Cellular and Molecular Pathology of Cardiovascular System, Institute of Human Morphology, 3 Tsyurupa Street, 117418 Moscow, Russia; 3 Institute of Carcinogenesis, N.N. Blokhin Cancer Research Center, 115478 Moscow, Russia; alexei.gratchev@gmail.com

In this Special Issue of Biomedicines, we have many insightful reviews and research papers on the subject “Macrophages in Health and Non-infectious Disease”, but first; we should discuss briefly the current situation in the field.

Macrophages are a highly heterogeneous group of cells with highly variable receptor profile and functional activities responsible for regulation and maintenance of multiple processes, including inflammation and innate immune response.

The chronic inflammation is a pathogenic mechanism of many diseases and the leading cause of morbidity and mortality in the world, especially in developed countries. The exact causes of uncontrolled inflammation are still to be discovered despite many recent advances in the field of innate immunity [1].

Innate immunity is a conserved mechanism of host defense against different types of pathogens and aggressive factors. Pathogen-associated molecular patterns (PAMPs) and damage-associated molecular patterns (DAMPs) serve as main inflammatory initiators. However, it should also be mentioned that, important for the initiation of atherosclerosis-related sterile inflammation, modified low-density lipoproteins (LDL), while belonging neither to PAMPs nor to DAMPs, are able to induce non-infectious inflammation. Every cell has organelles called mitochondria which can be the source of DAMPs (and thus stimulate innate immunity). When cells are damaged, mitochondrial DAMPs get released. Mitochondria are eukaryotic organelles having their origin from bacteria and carrying their own genome and some features of bacteria such as phospholipid cardiolipin in the mitochondrial inner membrane. The ability of defective mitochondria to induce immune response was shown due to similarity between bacteria and mitochondria on molecular level.

Inflammasomes are involved in a response to PAMPs and DAMPs stimuli, which results in the production of pro-inflammatory cytokines, IL-18 and interleukin-1β and cellular death. Mitochondria plays an important role in the initiation and regulation of NLRP3 inflammasome [2]. NLRP3 activators lead to the release required for inflammasome activation cardiolipin [3], mitochondrial reactive oxygen species and mitochondrial DNA. Activated inflammasomes lead to Caspase-1-dependent damage of mitochondria and blockage of mitophagy [4] which, in turn, stimulates immune response. Mitophagy is a type of autophagy and quality control system responsible for recognizing and degrading of damaged mitochondria using lysosomes [5]. Defects in mitophagy result in an accumulation of dysfunctional mitochondria and cytokine-induced inflammation [6]. There is a correlation between certain mtDNA mutations and atherosclerosis [7].

Our views on the mechanisms of chronification of inflammation are shown in Figure 1.

This Special Issue has many interesting discoveries and reviews which will be mentioned briefly below.

Kauerova with coauthors [8] investigated the influence of statins on macrophage polarization in a larger number of individuals than they did in their previous study. They analyzed data of two studies with the addition of an animal model, and the model of human macrophages to study the effect of statin in vitro. The authors confirmed that the pro-inflammatory macrophage proportion correlates with LDL cholesterol levels.

Vishnyakova with coauthors [9] compared expression of pro-inflammatory, anti-inflammatory markers, and estrogen receptor α in decidual macrophages in normal pregnancy and in patients with early- and late-onset preeclampsia. They discovered that the levels of HGF, CD206, and estrogen receptor α were downregulated in the case of preeclampsia, which may be the factor contributing to its pathogenesis.

Fiorelli with coauthors [10] evaluated Netrin-1, a laminin-like protein that plays a pivotal role in cell migration and exerts both pro- and anti-atherosclerotic functions, in spindle and round monocyte-derived macrophages (MDMs) from coronary artery disease (CAD) patients and control individuals. They found that the plasma levels of Netrin-1 were lower in CAD patients and the expression of Netrin-1 and its receptor in MDMs were higher in patients than in controls. The authors discovered that CAD patients with high intracellular Netrin-1 content also had a greater intraplaque macrophage accumulation which supported the idea about Netrin-1 involvement into atherosclerosis development.

Pireaux with coauthors [11] found that levels of inflammation biomarkers (M-CSF, CRP, and IL-8) in hemodialyzed patients were significantly increased, as well as the levels of myeloperoxidase-associated oxidative stress biomarkers. They also discovered that the plasma of patients experiencing a hemodialysis procedure had a higher content of M2 monocytes in comparison with controls.

Elchaninov with coauthors [12] studied the gene expression profile, immunophenotype, proteome, and pool of microRNA of Kupffer cells and monocytes. It was discovered that the observed differences did not allow to consider the resident liver macrophages as pure M2 macrophages. They found that monocytes demonstrated a high plasticity and sensitivity to molecular patterns associated with pathogens and that the resident liver macrophages were associated with the regulation of specific organ functions.

Martin-Cordero with coauthors [13] found that global phenotypic anti-inflammatory effects were caused in lean and obese sedentary mice upon β2-adrenergic activation. The effects were more pronounced (with the cytokine profile of anti-inflammatory effect) in obese animals. The anti-inflammatory effect was weaker in exercised lean and obese mice, and was accompanied only by the decreased expression of IL-8 and iNOS.

Melnikov with coauthors [14] studied the transport of monomeric and pentameric forms of C-reactive protein (mCRP and pCRP) by blood cells and their microparticles in the blood of patients with coronary artery disease. They found that monocytes were mostly pCRP-positive (93%) and 22% of monocyte-derived exosomes contained mCRP.

Kochumon with coauthors [15] summed their findings, creating a new model for the role of stearic acid in the pathologic production of MIP-1α/CCL3 in the presence of TNF-α associated with obesity.

Akata with coauthors [16] discovered the responsibility of non-polarized macrophages for the poor phagocytic capacity of lung macrophages in COPD resulting in reduced capacity for pathogen recognition and processing. It could contribute to the severity of the disease.

Lee with coauthors [17] found that the key regulators of low temperature-enhanced cancer progression in the tumor microenvironment are the processes of secretion and utilization of glutamine by macrophages and cancer cells.

Kovaleva with coauthors [18] showed that the combination of increased iNOS expression with high bacterial load in the tumor is a favorable prognostic factor, and the combination of the increased number of FOXP3+ cells with the high bacterial load is a poor prognostic factor. The authors found that the prognostic value of the bacterial load of the tumor depends on the status of local antitumor immunity.

Ara with coauthors [19] found that the systemic low-grade inflammation in post-obese men with a long-term weight loss was similar to non-obese control individuals despite a higher content of subcutaneous adipose tissue CD68^+^ macrophages. The authors also discovered that the content of anti-inflammatory CD163^+^ macrophages in adipose tissue was higher in post-obese men than obese men and non-obese men.

Rossi with coauthors [20] reviewed the dual effect of macrophages in the pathogenesis of ischemia-reperfusion injury-induced acute kidney injury and discussed the critical role of heme oxygenase-1-expressing macrophages.

Mesaros with coauthors [21] reviewed the current knowledge on the role of stromal macrophages in chronic lymphocytic leukemia.

Osman with coauthors [22] wrote a review about endothelial dysfunction and the roles of large extracellular vesicles (lEV) and endoplasmic reticulum (ER) stress in this process and also described the molecular interactions between lEVs and ER stress during endothelial dysfunction.

Skuratovskaia with coauthors [23] reviewed the idea that chronic inflammation is related to the accumulation of critically activated macrophages in one site with a focus on obesity. The authors analyzed the data from the scientific publications on macrophages and their involvement in the pathogenesis in different tissues during aseptic inflammation in obese individuals.

Sukhorukov with coauthors [24] discussed the mechanisms of formation of endoplasmic reticulum stress and the relationship between lipid accumulation and pro-inflammatory response. The authors specifically focused their attention on macrophages, which play an important role in the maintenance of chronic inflammation in atherosclerosis, and also contribute to the production of foam cells.

Yegorov with coauthors [25] described the approaches which could help to mitigate the negative effects related to oxidative stress. They discuss the use of antioxidants, activation of mitochondrial uncoupling, stimulation of mitophagy and expression of the telomerase catalytic component gene.

Kübler with coauthors [26] studied cold-inducible RNA-binding protein (CIRP) and its role in angiogenesis and regeneration of ischemic muscle tissue. They found that, when CIRP was absent, it helped with angiogenesis and regeneration of ischemic muscle tissue, and the mechanism of these processes is most probably related to the direction of macrophage polarization to regenerative M2-like macrophages.

In conclusion, we would like to express our hope that the high-quality standard of publications established in this Special Issue (SI) will be continued in the next version of SI.

## Figures and Tables

**Figure 1:**
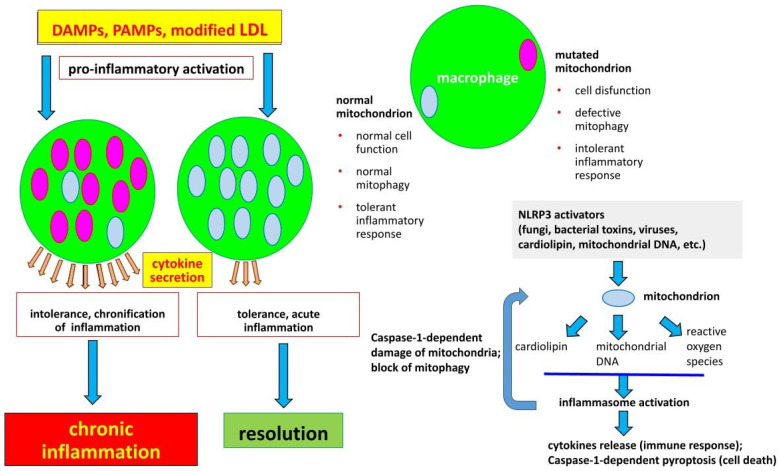
The development of chronic inflammation due to presence of damaged mitochondria in macrophages. A macrophage normally recognizes damage-associated molecular patterns (DAMPs), pathogen-associated molecular patterns (PAMPs), or modified (atherogenic) low-density lipoproteins (LDL) which results in a pro-inflammatory activation and release of cytokines. Normally immune tolerance gets developed, meaning that the macrophage will not respond to subsequent stimuli eventually leading to the resolution of inflammation. However, in the case of presence of certain mitochondrial mutations, macrophages lose their ability to develop immune tolerance, leading to an increased cytokine production after pro-inflammatory activation and the development of chronic inflammation. Mitochondria also regulate activation of NLRP3 inflammasome, a macromolecular complex consisting of nucleotide-binding domain leucine-rich repeat containing (NLR) family member NLRP3, that connects to Caspase-1 via apoptosis-associated speck-like protein containing a caspase recruitment domain (ASC). NLRP3 inflammasome is formed in response to DAMPs (including reactive oxygen species) and PAMPs (including cardiolipin and mitochondrial DNA) and induces the release of pro-inflammatory cytokines and pyroptosis (cellular death) [2,3,4,5,6,7].
